# Shell isolated nanoparticle enhanced Raman spectroscopy for mechanistic investigation of electrochemical reactions

**DOI:** 10.1186/s40580-022-00301-1

**Published:** 2022-02-14

**Authors:** Andi Haryanto, Chan Woo Lee

**Affiliations:** Department of Chemistry, Kookmin University, Seoul, 0207 South Korea

**Keywords:** Electrocatalysis, In situ spectroscopy, Raman scattering, Reaction mechanisms, Shell-isolated nanoparticle-enhanced Raman spectroscopy

## Abstract

Electrochemical conversion of abundant resources, such as carbon dioxide, water, nitrogen, and nitrate, is a remarkable strategy for replacing fossil fuel-based processes and achieving a sustainable energy future. Designing an efficient and selective electrocatalysis system for electrochemical conversion reactions remains a challenge due to a lack of understanding of the reaction mechanism. Shell-isolated nanoparticle-enhanced Raman spectroscopy (SHINERS) is a promising strategy for experimentally unraveling a reaction pathway and rate-limiting step by detecting intermediate species and catalytically active sites that occur during the reaction regardless of substrate. In this review, we introduce the SHINERS principle and its historical developments. Furthermore, we discuss recent SHINERS applications and developments for investigating intermediate species involved in a variety of electrocatalytic reactions.

## Introduction

Environmental deterioration has become a major concern around the world. Energy-generating processes are currently reliant on fossil fuels, which emit greenhouse gases, such as carbon dioxide (CO_2_), methane, and nitrous oxide [1–3]. Based on the British Petroleum Statistical Review of World Energy in 2020, fossil fuels accounted for 84.3% of the overall energy consumption in 2019. In line with this, CO_2_ emissions increased by 0.5% in 2018, reaching 34,169 million tons [4]. Accumulated CO_2_ molecules in the atmosphere primarily contribute to environmental deterioration by lowering seawater pH and causing global warming through the greenhouse effect [5].

Replacing fossil fuels with renewable energy sources, such as hydropower, wind, and solar, as well as directly converting CO_2_ into other chemicals using renewable energy sources, have all proven to be effective strategies for mitigating CO_2_ emissions. Hydrogen is another promising candidate for replacing fossil fuels because of its ability to transport energy [6]. Chemical energy is converted to electricity in a fuel cell via hydrogen oxidation reaction and oxygen reduction reaction (ORR). Moreover, renewable energy sources can be used to obtain green hydrogen via a water-splitting reaction [7]. These approaches have been studied and tested as a possible solution to environmental problems.

It is important to investigate the reaction pathway and rate-determining step (RDS) in environmentally friendly energy conversion processes, where essential intermediate species are involved in the reaction. Researchers could develop new ideas based on the RDS to produce a superior electrocatalyst with optimal binding strengths for the key intermediates [8]. For example, OOH* and CO* intermediates are involved in the electrochemical reduction of CO_2_ to CO. When the COOH* binding strength is extremely weak to be adsorbed at a specific surface, increasing the binding strength can improve catalytic performance by facilitating the RDS [9, 10].

In situ surface-enhanced Raman scattering (SERS) has been used to investigate the reaction mechanisms of various electrochemical reactions, including hydrogen evolution reaction (HER) [11], oxygen evolution reaction (OER) [12], ORR [13], and CO_2_ reduction reaction (CO_2_RR) [14]. Raman measurement collects the inelastic photon scattered from the excitation of vibrational modes within the sample and at the surface. It can detect catalyst phase evolution and intermediate species adsorbed on the catalyst surface. In situ SERS allows for detection of more surface vibrations in a broader spectral range, such as metal–oxygen and metal–carbon in the low wavenumber region, in comparison with in situ attenuated total reflection surface-enhanced Infrared absorption spectroscopy (ATR-SEIRAS) [15, 16]. In addition, the prisms used as a substrate in ATR-SEIRAS impose limitations in terms of electrolyte pH and available wavenumber range [17], while SERS is measured on an actual catalyst electrode. Furthermore, analysis of SERS spectra is straight-forward because background subtraction is not needed as in ATR-SEIRAS [18]. However, the catalyst surfaces that can be investigated by SERS are mostly Au, Ag, and Cu metals that have an intrinsic ability to facilitate surface plasmon resonance and amplify Raman signals generated at the surface. Other metals, such as Pd, Pt, Ni, and Co, have also been investigated, but the Raman enhancement was relatively low, and the adsorption of the chemical species on catalyst surfaces was difficult to detect [19].

To overcome the limitation, shell-isolated nanoparticle-enhanced Raman spectroscopy (SHINERS) has been proposed as a general method for enhancing Raman signals from surface intermediate species in electrocatalysis reactions. The plasmonic core in SHINERS is coated with a thin protective layer, which prevents direct interaction between a probe molecule and the plasmonic core while allowing for surface plasmon resonance. The shell-isolated nanoparticle (SHIN) is then placed on the target substrate to detect molecules adsorbed at the surface of a substrate. In this regard, it has been reported that this method can be applied to any kind of substrate [20]. In addition, the shell can improve the plasmonic core stability in a wide range of pH and electrolytes [21]. Here, the historical development and fundamental principle of SHINERS technology are discussed. Next, recent advances of SHINERS in investigating reaction mechanisms of electrochemical reactions, such as ORR, CORR, CO electrooxidation reaction, OER, and nitrate reduction reaction (NO_3_RR), are outlined. Finally, the potential application of SHINERS in other electrocatalysis fields is discussed.

## Principle of SHINERS

**Fig. 1:**
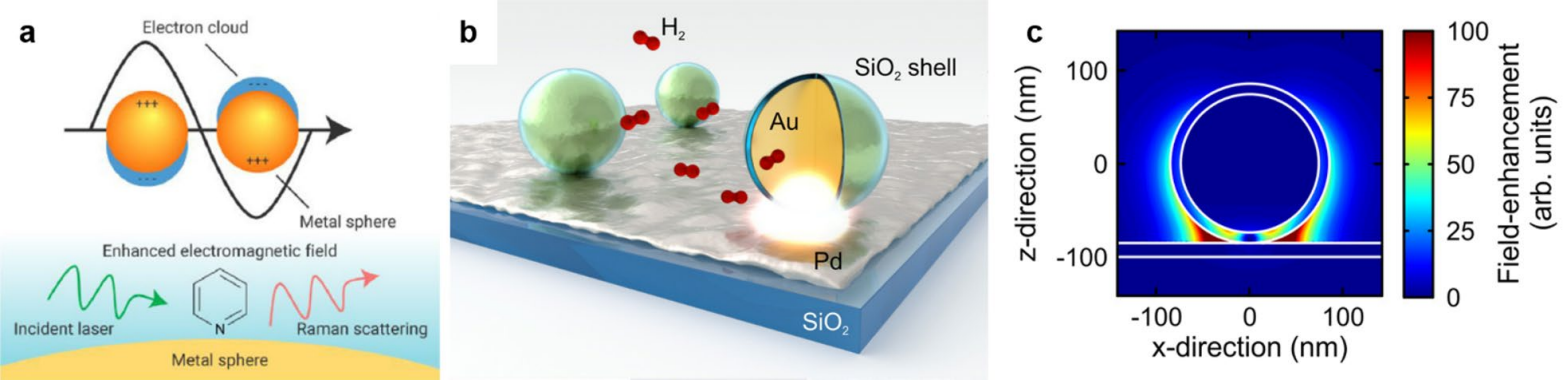
**a** Illustration of the localized surface plasmon resonance on the nanoparticle surface, reproduced with permission from [24]. Copyright (2020) Elsevier B.V. **b** SERS measurement at the interface of the core–shell nanoparticle and the electrode. **c** The distribution of field-enhancement around the surface of a core–shell nanoparticle, reproduced with permission from [23]. Copyright (2013) American Chemical Society

The enhancement in SERS mainly originates from the localized surface plasmon resonance (LSPR). The LSPR is a special phenomenon that occurs by collective coherent oscillating modes of electrons on nanoparticle surfaces under light illumination, as illustrated in Fig. 1a. The oscillating mode causes localized electromagnetic fields around the nanoparticle surface, which contribute to the enhancement of Raman intensity [22–24].

The gap distance between each nanoparticle also affects the SERS enhancement factor. The local electromagnetic field in the nanogap between two or more nanoparticles is strong because of the electromagnetic coupling of the nanoparticles. According to Tian’s group, the SERS enhancement factor of Au nanosphere dimer increased by 10,000 times from 10^5^ to 10^9^ when the gap distance reduced from 10 to 2 nm [22]. However, reducing the nanogap between each nanoparticle has its limitation since electron quantum tunneling effects between the coupled nanoparticles will occur. The electron tunneling, e.g., charge transfer across the narrow junction in the sub-nanometer level, decreases the electric field across the junction and thus causes a considerable reduction in the electromagnetic field enhancements across the dimer junction [25].

At the interface of the core–shell nanoparticle and the plasmonically inactive electrode (Fig. 1b and c), the field enhancement is more intensified around the contact between the core–shell nanoparticle and the electrode film. Under light illumination, the core–shell nanoparticle induces a mirror plasmon oscillation from the electrode [26]. The coupling of those oscillating plasmon results in a high electromagnetic field concentrated around the contact point, which further contribute to the amplification of Raman intensity from probed molecules.

**Fig. 2:**
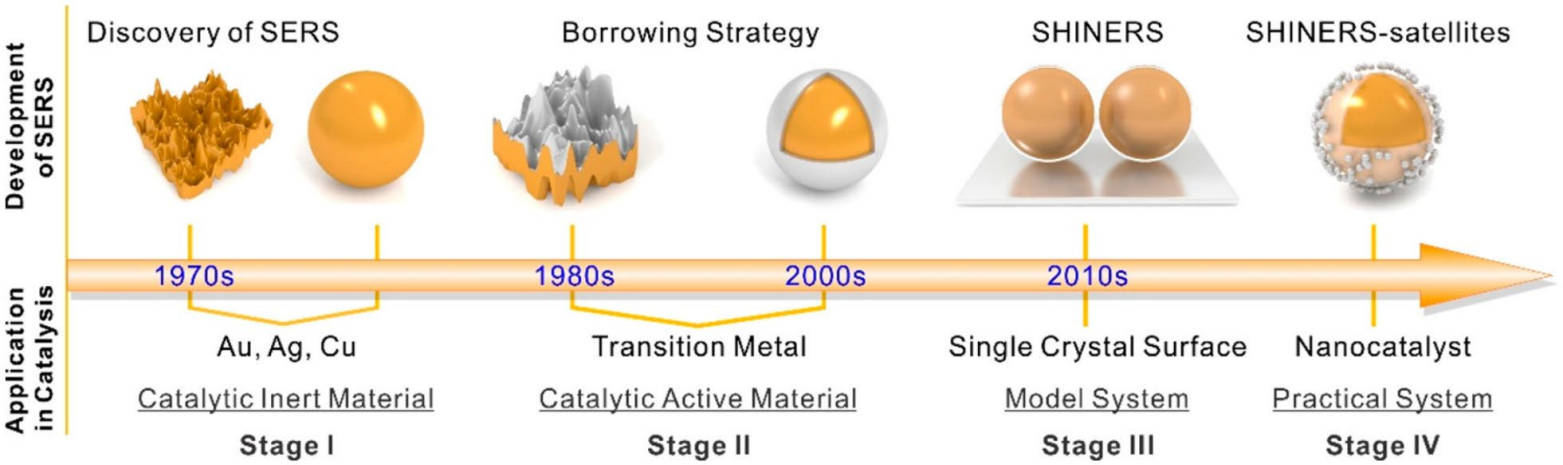
The historical illustration of surface-enhanced Raman spectroscopy for the catalysis application. Discovery of SERS (stage I), borrowing strategy for non-plasmonic active materials (stage II), application of SHINERS on single crystalline surface as a model system (stage III), and practical system of SHINERS with satellite structure of nanocatalysts (stage IV), reproduced with permission from [27]. Copyright (2020) American Chemical Society

Figure 2 shows the historical progress on SERS application in catalysis, from the discovery of the SERS effect to the borrowing strategy, SHINERS method, and SHINERS satellite structures. In 1974, Fleischmann et al. discovered that the Raman signal of adsorbed pyridine was significantly enhanced on electrochemically roughened silver electrodes [28]. Later on, in 1977, Van Duyne et al. reported that the Raman signal from pyridine adsorbed was about 10^5^–10^6^ times stronger than that of bulk pyridine [29]. The researchers initially concluded that the significant enhancement of the Raman signal was due to an increase in the amount of pyridine absorbed at the electrode surface. However, it was later revealed that the enhancement was due to the localized electromagnetic field formed around the metallic nanostructures [19].

After the SERS effect findings, many researchers have explored different types of catalysts for Raman signal enhancement [19]. However, only Ag, Au, and Cu metals have been found to have high enhancement factors of Raman signals because these metals intrinsically generate a large electromagnetic field around the metal nanoparticle surface [21, 30]. To overcome the limitation and probe various catalyst surfaces in addition to Ag, Au, and Cu, a borrowing strategy has been utilized [27]. As shown in Fig. 2, the objective was to deposit a thin layer of the catalytically active material of interest, represented by silver color, on the yellow-colored SERS-active material. As a result, the catalyst surface can borrow a localized electric field from the under layer. Despite the utility of the borrowing strategy, obtaining vibrational information of adsorbed molecules on atomically flat surfaces, such as various single-crystalline electrodes, which are widely used as a model system in surface science and electrochemistry, was difficult because flat electrodes are relatively inactive for SERS [27].

**Fig. 3:**
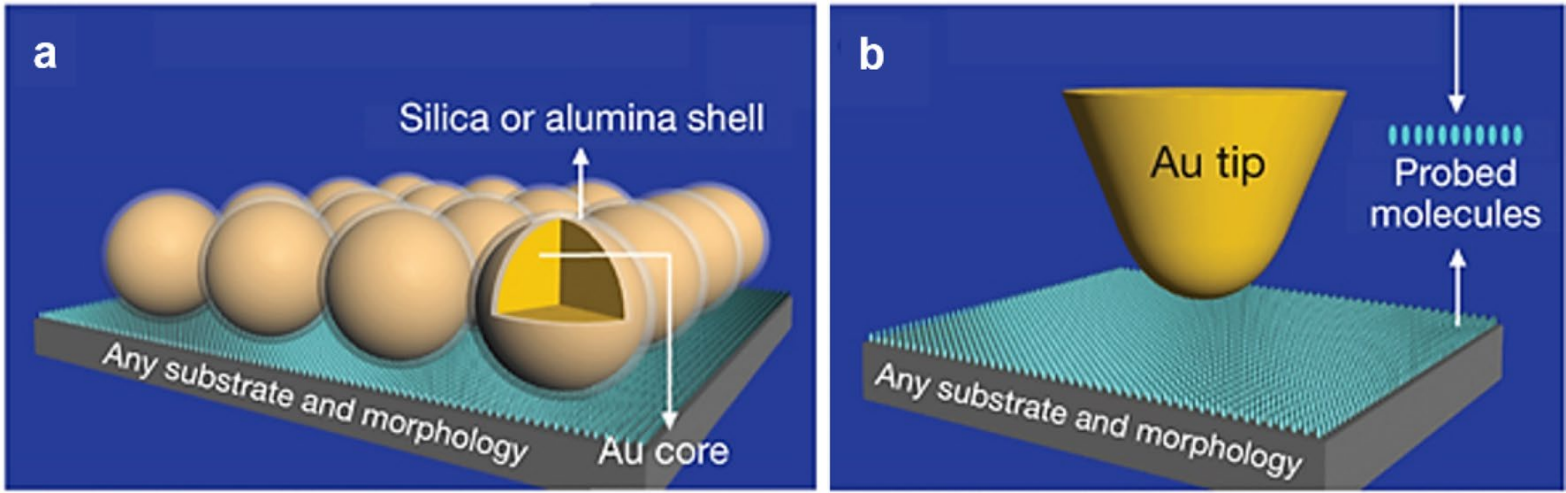
The comparison between SHINERS working principle with other **a** shell-isolated mode: SHINERS. **b** non-contact mode: tip-enhanced Raman spectroscopy, reproduced with permission from [21]. Copyright (2010) Macmillan Publishers Limited

Tian et al. developed SHINERS in 2010 to solve the limitations of the catalyst used in SERS [21]. Figure 3a shows the SHINERS electrode structure and working principle. They synthesized Au nanospheres coated with pinhole-free and ultrathin silica (or alumina) shells. The Au/SiO_2_ core/shell nanoparticles were coated onto the substrate adsorbed by probed molecules. The principle is the same as that of tip-enhanced Raman spectroscopy (TERS), in which the Au tip end can provide localized electromagnetic field enhancement, and the probed molecule can borrow the enhancement from the Au tip, as shown in Fig. 3b. In SHINERS, the core–shell nanoparticles act as the TERS tip. The layer of nanoparticles spread over the catalyst electrode provides many TERS tips, resulting in a very strong Raman signal [19, 21, 31]. Furthermore, since silica shell is usually more inactive than the Au surface for many catalytic reactions and intermediate adsorption [21], it can act as a protective layer for the conservation of SERS-active Au core and the inhibition of the interaction between Au and probed molecules, which is a great advantage of the SHINERS method.

**Fig. 4:**
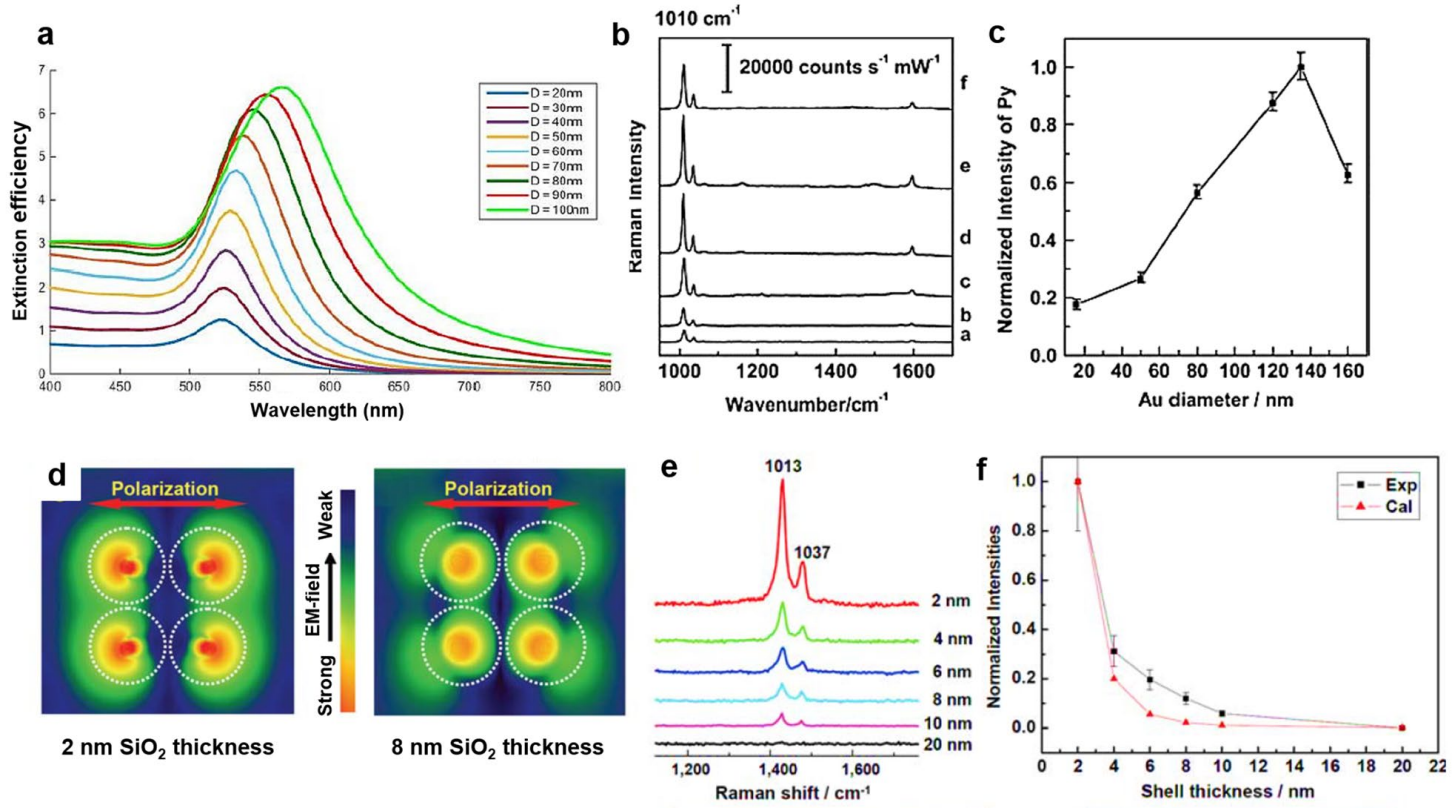
**a** The extinction efficiency of Au nanospheres with different diameters, reproduced with permission from [32]. Copyright (2018) IOP Publishing Ltd. **b**) SERS spectra of pyridine adsorbed on Au nanoparticles with different sizes: 16 to 160 nm (from **a**–**f**). **c** The normalized SERS intensity of pyridine as a function of the Au nanoparticle size, reproduced with permission from [33]. Copyright (2008) John Wiley and Sons, Ltd. **d** The top view of 3D-FDTD simulation from a 2 × 2 array of 55 nm Au@SiO_2_ nanoparticles showing the distribution of electromagnetic field enhancement as a function of shell thickness (the color gradient represents the strength of the electromagnetic field, and the white dash circles indicate the Au@SiO_2_ nanoparticles. **e** SHINERS spectra of pyridine adsorbed on a smooth Au surface coated with Au@SiO_2_ with different silica shell thicknesses. **f** The dependence of the normalized intensity of pyridine on the shell thickness, reproduced with permission from [21]. Copyright (2010) Macmillan Publishers Limited

The SHINERS activity is determined by the size of the nanoparticle core and the thickness of the shell. Different sizes of nanoparticles have different extinction spectra and resonance wavelengths under light illumination, which affects the Raman enhancement factor, as shown in Fig. 4a [32, 34, 35]. Fang et al. evaluated the SERS activity of Au nanoparticles with sizes ranging from 16 to 160 nm that were adsorbed using pyridine as the probe molecule in the absence of a silica shell [33]. Figure 4b shows the SERS spectra of pyridine adsorbed on Au nanoparticles of different sizes. At a diameter of 135 nm, the SERS intensity of v_1_ pyridine vibration positioned at 1007 cm^−1^ was at its maximum (approximately 32,000 counts s^−1^ mW^−1^). Figure 4c shows the relationship between SERS intensity and nanoparticle size. These results show that there is an optimum nanoparticle size that is consistent with the 633 nm laser excitation wavelength. The resonance wavelength of SERS is widely known to be dependent on the nanoparticle size [36]. Shell thickness also affects the Raman signal enhancement in SHINERS. As shown in the three-dimensional finite-difference time-domain (3D-FDTD) simulation result from a 2 × 2 array of 55 nm Au@SiO_2_ on Au substrate (Fig. 4d), the distribution of electromagnetic field enhancement changes with the thickness of the SiO_2_ shell [21]. A common trend that electromagnetic field decreases with distance from nanoparticle is observed for 2 and 8 nm SiO_2_ thicknesses while stronger field occurs on a broader space in the case of 2 nm SiO_2_ by LSPR [19]. When the SiO_2_ shell becomes thinner, the distance between the Au core-Au core and Au core-Au substrate decreases. Therefore, the LSPR can be occurred on the junction between Au core-Au core and Au core-Au substrate, resulting in stronger electromagnetic field enhancement. As a result, the Raman peak intensity of pyridine adsorbed on the smooth Au@SiO_2_-coated Au substrate decreases as the SiO_2_ shell thickness increases (Fig. 4e). Tian’s group also plotted the peak intensity of the Raman vibration positioned at 1013 cm^−1^ (normalized for the signal intensity obtained at 2 nm thickness) against the shell thickness (Fig. 4f) [21]. This result shows that a thin silica layer is preferred for use in the local electric field from the Au core, provided that a pinhole-free silica layer can be grown.

## Synthesis of SHINs

**Fig. 5:**
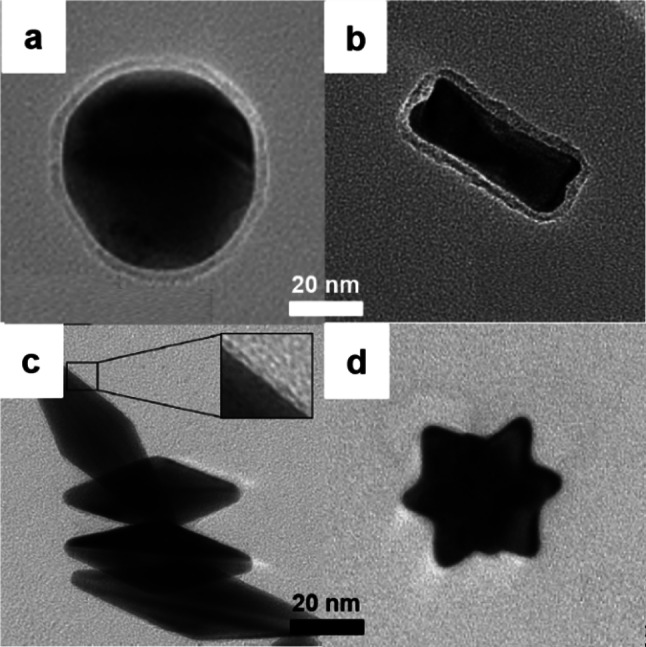
Different SHIN shapes. **a** Nanosphere, reproduced with permission from [21]. Copyright (2010) Macmillan Publishers Limited. **b** Nanorod, reproduced with permission from [37]. Copyright (2011) Oldenbourg Wissenschaftsverlag. **c** Nanodipyramid, reproduced with permission from [38]. Copyright (2018) Elsevier. **d** Nanostar, reproduced with permission from [39]. Copyright (2019) Elsevier

Since SHINERS was developed, many researchers have tried to examine different shapes of SHINs to further improve the SERS activity of SHINERS. As shown in Fig. 5, nanospheres, nanorod, nanostar, and dipyramids are the synthesized sample shapes. Additionally, different core (Au and Ag) and shell (SiO_2_ and Al_2_O_3_) materials were used. In this chapter, various synthetic strategies for preparing different SHINs are explained in detail.

The spherical nanoparticle is the most commonly used shape for SHINERS because of its simple preparation. The Au nanospheres are generally synthesized using the method developed by Turkevich and Frens [40, 41]. In the synthetic method, HAuCl_4_ solution was first heated, and sodium citrate solution was subsequently added. After the solution color changed to red, the heating was continued for 30 min, and the resultant solution was cooled to room temperature [40]. Sodium citrate acts as a reducing agent and a stabilizing agent to produce monodisperse and uniform Au nanoparticles. Nanosphere particle size can be controlled by adjusting the amount of sodium citrate. If the amount of sodium citrate is reduced, the size of the nanosphere would be small [41].

Nanorods have been widely used in SERS activity control [37]. Au nanorod was first synthesized based on a method by Sau and Murphy [42]. The HAuCl_4_ was mixed with cetyltrimethylammonium bromide (CTAB), which served as a shape-controlling agent for the gold nanoparticle. Next, NaBH_4_ solution, a reducing agent, was added while stirring, and the solution had a pale brown-yellow color. The seed solution was then added to the growth solution consisting of AgNO_3_, CTAB, and HAuCl_4_. The Ag^+^ ions serve as sacrificial ions, allowing for the reduction of Au^3+^ ions to form Au nanorods. Afterward, (3-aminopropyl)trimethoxysilane (APTMS) solution was added to the nanorod solution while stirring, and sodium silicate was added for SiO_2_ layer coating [37]. Quyen et al. demonstrated that the Raman intensity of rhodamine 6G captured with a rod-shaped Au@SiO_2_ as the SERS material was more than 3 times higher than that captured with spherical-shaped Au@SiO_2_ [43]. The signal enhancement was attributed to the different photophysical property of Au@SiO_2_ nanorods. It had a long-wavelength band in the near-infrared region with a high absorption intensity.

Sharp edges are known for increasing the electromagnetic field surrounding plasmonic materials because of their high electron density [51–54]. Kudelski’s group developed dipyramidal-shaped Au@SiO_2_ nanoparticles to increase the efficiency of Raman signal enhancement in SHINERS [38]. Dipyramidal gold was synthesized by growing pyramids on gold nanoparticle seeds with two key factors: pH and Ag^+^ concentration. A solution with a low pH is required to increase the redox potential of ascorbic acid. In this condition, an Ag monolayer can be deposited on the gold nanoparticle surface, stabilizing it and inhibiting tip growth. When using a high pH, the Au^3+^ reduction rate increased, resulting in fast deposition along the crystal face and short aspect ratio nanorod development in all directions [55]. Raman spectra of 4-mercaptobenzoic acid (MBA) and 2-mercaptoethanesulfonate (MES) were examined on a platinum surface covered with dipyramidal shaped Au nanoparticles and spherical shaped Au nanoparticles for Raman intensity enhancement. In comparison with spherical-shaped Au nanoparticles, dipyramidal-shaped Au showed a significant enhancement in Raman intensity of about 11 and 16 times for MBA and MES, respectively.

In a similar context, Krajczewski et al. synthesized a star-shaped silica-coated gold nanoparticle with a higher number of edges [39, 56]. They used HAuCl_4_ as a gold precursor, H_2_O_2_ as a reducing agent, and NaOH to trigger the reduction reaction and stabilize the gold nanostar nanoparticle. Tetraethyl orthosilicate (TEOS) was used as a silica source for SiO_2_ coating. The SiO_2_ shell growth was controlled by the adjusting reaction time; a 15-min reaction time resulted in a 2–4 nm thick layer. The same group also compared the Raman enhancement efficiency of MBA monolayers on the Pt surface covered with Au nanostar and spherical Au nanoparticles using SERS spectra. The Raman spectra obtained by MBA using Au nanostar as SERS probe were enhanced more than 10 times when compared to spherical Au nanoparticles [39].

Although Au metal is widely used as a core nanoparticle in SHINERS, other metals with high plasmonic characteristics, such as Ag, can also be used. Tian’s group synthesized Ag nanospheres with Au seeds because Ag has a tendency to be oxidized, and this resulted in the formation of anisotropic nanoparticles [57]. Ascorbic acid was used to reduce the Ag^+^ ions on the Au seed surfaces. The polyol method has also been used to synthesize silver nanowires. In this method, ethylene glycol is used as a solvent and reducing agent. Polyvinylpyrrolidone acts as a capping agent, regulating the growth rate of silver nanowires [58]. Although Ag is more easily oxidized and mobile than Au, it is significantly less expensive and has a higher surface plasmon efficiency than Au. Tian et al. observed that the enhancement effect of the pyridine Raman intensity in Ag@SiO_2_ nanoparticles was 100 times higher than that in Au@SiO_2_ [57].

The plasmonic core in the SHINERS was coated with an ultrathin metal oxide shell, such as SiO_2_ and Al_2_O_3_. For silica coating, APTMS solution was added to the solution of Au or Ag nanoparticles under vigorous stirring. The APTMS caused the Au nanoparticle surface to generate silanol functional groups, which are responsive to silicate ion deposition [59, 60]. Subsequently, a sodium silicate solution with a pH of 10.2 was added to the mixture. One of the important parameters for SiO_2_ shell growth is pH regulation. If the pH of sodium silicate is higher than 11, shell dissolution by NaOH will obstruct its growth, resulting in the detection of a pinhole. If a lower pH (pH 8) is used, the SiO_2_ shell will rapidly grow and become excessively thick.

The reaction temperature is another key parameter that must be controlled. Tian’s group used a 90 °C water bath for SiO_2_ shell growth instead of the typically used room temperature [21]. The elevated temperature can reduce the reaction time from 48 or 72 h to just 1 h for producing SiO_2_ with a 4 nm shell thickness. The thickness of the silica shell can be delicately adjusted by controlling the reaction time. For example, a 2 nm shell thickness can be achieved by heating the mixture for 20 min, whereas thicker shells of 4 and 6 nm can be obtained by extending the reaction time to 1 and 2 h, respectively.

The atomic layer deposition (ALD) method can also be used to prepare ultrathin SHIN shells. In summary, the ALD method is used to coat Au core nanoparticles by sequentially purging the shell precursor gas, resulting in layer-by-layer growth with high uniformity. This method has been widely used to fabricate metal oxide shells, such as Al_2_O_3_ and TiO_2_ shells, which can be ultrathin and uniform without a pinhole [61]. Zhang et al. used the ALD method to obtain a large quantity of SHINs (1–10 g) with uniform shells by floating metal nanoparticles in a chamber via gas flow [62].

Noble metals such as Pt, Pd, Rh, and Ru are known to be inactive for SERS but catalytically active. Coating those metals on the plasmonically-active material also can be a way to probe the catalyst surface by constructing a plasmonic-catalytic hybrid nanoreactor. Acharya et al*.* applied plasmon-induced photocatalytic ALD to deposit atomically thin catalytic-active metal on the Au nanorod surface [63]. Firstly, the gold nanorod was individually isolated with a hollow silica shell. For catalytically-active metal coating, the hollow silica-coated Au nanorod dispersion was mixed with metal salt in ethylene glycol (EG) and irradiated with a near-infrared (NIR) laser with a power density of 0.4 W/cm^2^ at 35 °C. EG was used to control the metal shell growth around the Au nanorod surface. If stronger reducing agents such as ascorbic acid or hydroxylamine were used, a dendritic shape metal growth would be resulted instead of the conformal metal coating.

## SHINERS application

SHINERS has been used to gain mechanistic insights into various electrochemical reactions, including HER [21, 44], ORR [20, 48], CO reduction reaction (CORR) [15, 47], CO oxidation reaction (COOR) [64, 65], CO adsorption [44], OER [46], and NO_3_RR [50]. Table 1 summarizes the applications of SHINERS in the electrocatalysis research field, as well as the detailed experimental conditions, such as SHINs, electrode, electrolyte, and laser wavelength. According to previous studies, SHINERS has proven to be a powerful method for probing intermediate species that are rarely detected. For example, SHINERS can be used to monitor the vibrational modes of the binding of an atom and a smooth metal electrode with a low SERS activity (e.g., Pt(111)–H and Rh(111)–H) regardless of the type of metal substrate [21, 44]. The vibrational information obtained by SHINERS could be used to provide further insight into enhanced activities of high-performance electrocatalysts, provided that the binding strength of key intermediates at the catalyst surface has a strong correlation with the catalytic activity based on the Sabatier principle [9, 66]. Furthermore, SHINERS can be applied to a particular metal oxide film, such as electrodeposited iridium oxide (IrO_x_) film [46]. This study demonstrated a highly sensitive detection of an Ir = O stretching mode near the onset potential of the OER. In this regard, SHINERS has great potential for the mechanistic investigation of electrocatalysis, and its applications should be discussed in detail. In this chapter, we discuss several case studies in which the SHINERS technique was used to observe surface chemical species for ORR, CORR, COOR, OER, and NO_3_RR. The proposed reaction mechanisms and reaction pathways are discussed in detail based on the SHINERS results.

**Table 1:** Summary of reported applications and experimental conditions of SHINERS for electrocatalyst

Ref.	Core metal	Shell	Electrode	Electrolyte	Laser wavelength (nm)	Vibrational mode	Wavenumber (cm^−1^)	Application
[21]	Au	SiO_2_	Pt(111)	0.1 m NaClO_4_	632.8	Pt–H	2023	H adsorption
		Al_2_O_3_						
[44]	Au	SiO_2_	Rh(111)	0.1 m NaClO_4_	633	Rh–H	1897	H adsorption
[45]	Au	SiO_2_	Pt (111)	0.1 m HClO_4_ saturated by CO	632.8	Pt–CO	2071	CO adsorption
			Pt polycrystalline					
[46]	Au	SiO_2_	IrOx	1 m NaClO_4_ (pH 10)	632.8	Ir = O stretching	813	OER
	Au	SiO_2_	Cu foil	0.01 m KOH and 0.046 m K_2_SO_4_ (pH 11.7)	632.8			CO reduction
			Cu microparticles			Cu–O_(ad)_	612–622	
[15]			Cu Nanoparticle			Cu–CO_(bridge)_	1863–1873	
			Chem-Cu			Cu–CO_(top)_	2053–2070	
			Oxide derived Cu					
[47]	Au	SiO_2_	Cu dendritic microparticles	0.3 m NaH_2_PO_4_ and 0.35 m Na_2_HPO_4_ (pH 7.2)	632.8	CuO_x_/(OH)_y_	525–532	CO reduction
				0.1 m NaOH and 0.9 m NaClO_4_ (pH 12.9)		Cu–phosphate	931, 1124–1152	
[48]	Au	SiO_2_	Pd	0.1 m TBAClO_4_ in DMSO saturated by O_2_	785	O–O − in LiO_2_	1125	ORR
			Pt			C–O_2_ − in CO_2_Li	1500	
[49]	Au	SiO_2_	Pt(100) Pt(111)	0.1 m HClO4	632.8	Pt–O_2_ stretching O–O^−^ O–O^2−^	240 970 1200	Pt electrooxidation
[50]	Au	SiO_2_	Cu(100)	0.1 m HClO_4_ and 0.05 m HNO_3_ 0.1 m HClO_4_, 0.05 m HNO_3_, and 10 mm HCl	632.8	Cu_2_O in Cu(110) Cu_2_O in Cu(110)	509, 619 502, 621	Nitrate reduction
			Cu(110)					
			Cu(111)					
[20]	Au	SiO_2_	Pt(100)	0.1 m HClO_4_ saturated by O_2_ 0.1 m NaClO_4_ saturated by O_2_ (pH 10.3)	637.8	Pt–COOH Pr–OH	1005 1090	ORR
			Pt(110)					
			Pt(111)					

## Oxygen reduction reaction

The ORR is a cathodic reaction in fuel cells. The ORR catalyst must remain stable under extremely corrosive conditions [67]. Platinum-based nanocatalysts have been studied as ORR catalysts, but the structural effect of Pt-based catalysts on the ORR mechanism has not been well understood based on experimental results [68].

**Fig. 6:**
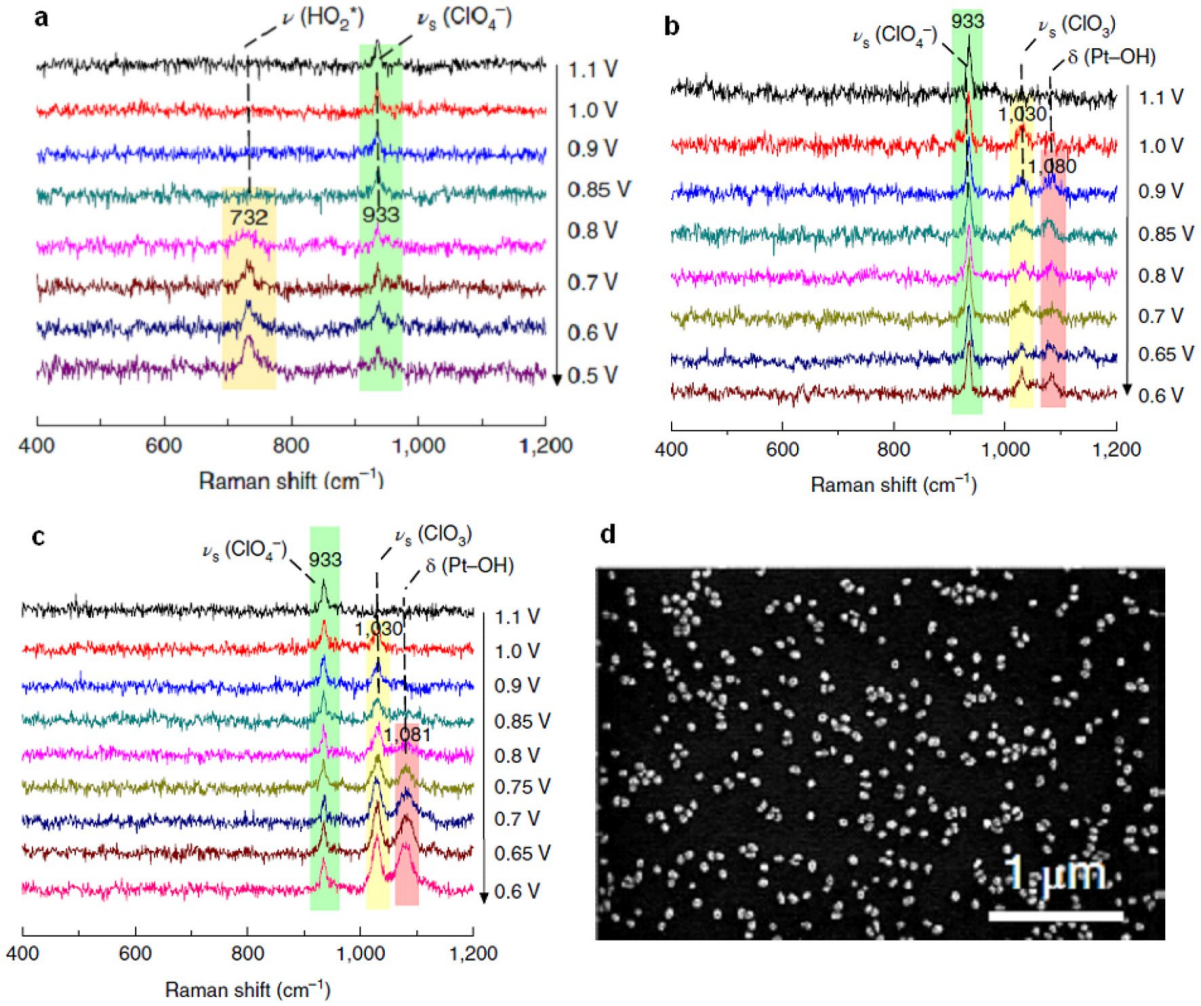
SHINERS of the ORR in a 0.1 M HClO_4_ solution saturated with O_2_ at the **a** Pt(111) electrode surface, **b** Pt(100) electrode surface, and **c** Pt(110) electrode surface, **d** The scanning electron microscopy (SEM) images of a Pt(111) single-crystal electrode surface modified with Au@SiO_2_, reproduced with permission from [20]. Copyright (2018) Springer Nature Limited

Dong et al. investigated the ORR process at Pt(hkl) surfaces in both acidic and alkaline conditions [20]. The direct evidence of intermediate species that occurred during the ORR process was captured using the SHINERS method. Figure 6a shows the SHINERS spectra of the ORR with the Pt(111) electrode obtained in acidic conditions on a potential range of 1.1–0.5 V. There was no Raman signal peak recorded above 0.8 V, except for the symmetric stretching mode of ClO_4_^−^ ion at 933 cm^−1^. A Raman peak around 732 cm^−1^ appeared at 0.8 V and gradually increased as the potential reached 0.5 V. The peak was assigned to the O–O stretching vibration of OOH* based on isotopic substitution experiments and density-functional theory (DFT) calculations. Conversely, the Raman spectra were noticeably different in the case of the Pt(100) and (110) surfaces. There were two Raman peaks recorded at 1030 and 1080 cm^−1^, which were assigned to the vibrational modes of ClO3 and OH*, respectively (Fig. 6b and c). The SEM image of the SHINs-modified electrode surface used in the experiment is shown in Fig. 6d.

According to previous studies, several intermediates, such as O2*, OOH*, O*, and OH*, are involved in ORR via four coupled proton-electron transfers, as shown below [12, 22]:

1 $${\text{O}}_{2} + * \to {\text{O}}_{2} *$$

2 $${\text{O}}_{2} * + {\text{H}}^{ + } {\text{e}}^{ - } \to {\text{OOH}}*$$

3 $${\text{OOH}}* + {\text{H}}^{ + } + {\text{e}}^{ - } \to {\text{O}* + \text{H}}_{2} {\text{O}}$$

4 $${\text{O}* + \text{H}}^{ + } + {\text{e}}^{ - } \to {\text{OH}}*$$

5 $${\text{OH* + H}}^{ + } + {\text{e}}^{ - } \to {\text{H}}_{2} {\text{O + *}}$$

The dissociation reaction of OOH*, in particular, is known as the rate-limiting step. The OOH* species was not stable on the Pt(110) and Pt(100) surfaces because it dissociated easily into O* and OH*, which was also supported by DFT calculations [20]. Therefore, the difference in the ORR activity between the Pt(111) and Pt(110) and (100) surfaces can be attributed to the difference in the OOH* dissociation. The Tian group also investigated the ORR process on Pt (211) and (311) surfaces with different applied potentials. They discovered two peaks that appeared at 786 and 1042 cm^−1^ after the potential reached 0.9 V and further confirmed that the two peaks were from OOH* and OH*, respectively, demonstrating that high-index facets of Pt metals have different intermediate adsorption features [69].

## Carbon monoxide reduction reaction

CO is an important chemical that is used as a precursor in the pre-existing petrochemical industry and recent electrochemical conversion research because of its ability to be converted into valuable hydrocarbons and alcohols, such as methane, methanol, and ethanol. Copper is a promising candidate for the electrochemical reduction reaction of CO, but the surface chemical species of Cu electrodes and its role in enhancing the CORR are still unclear [15].

**Fig. 7:**
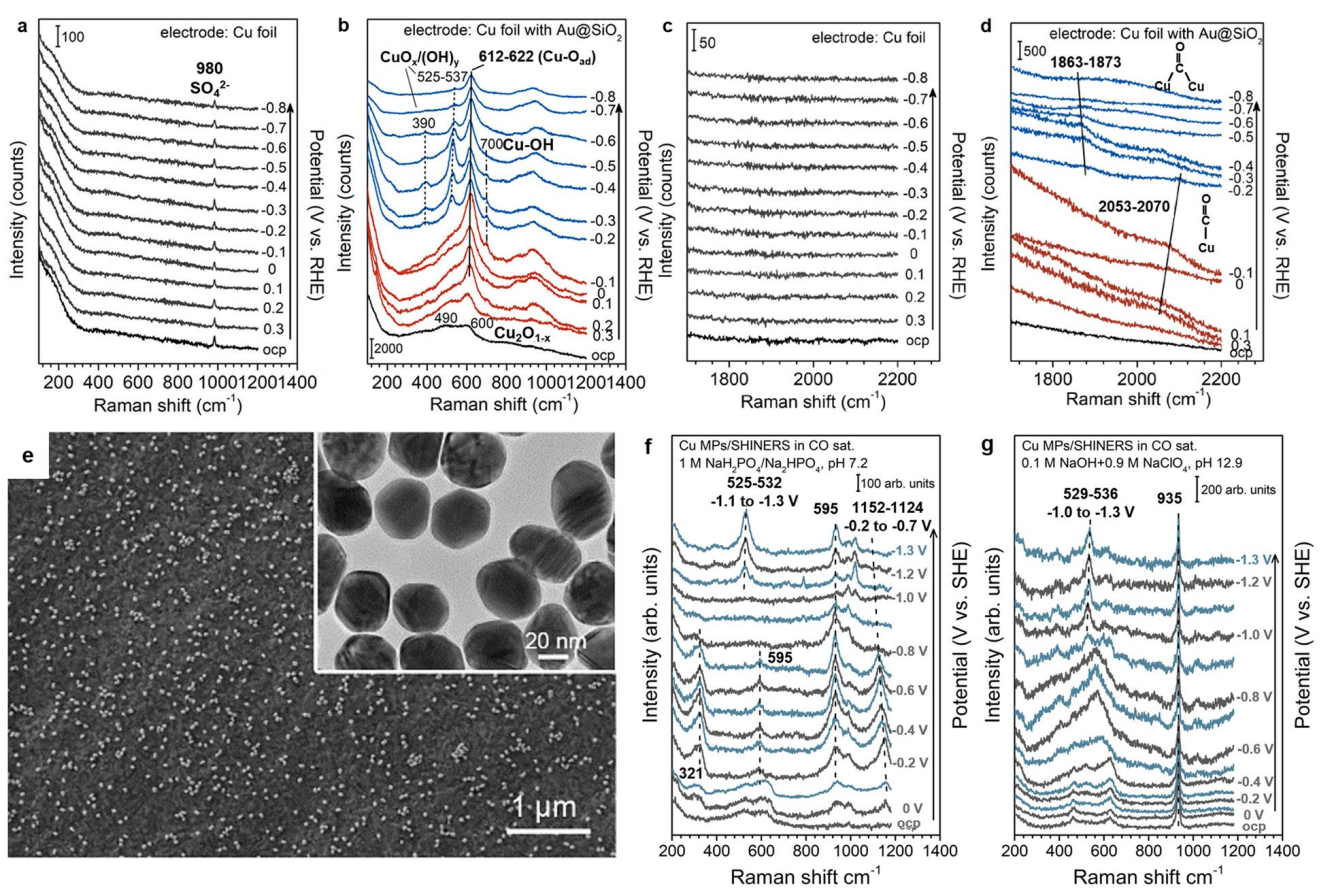
In situ Raman spectra measured at various potentials on **a**, **c** a Cu Foil and **b**, **d** a Cu foil with Au@SiO_2_. The measurement was conducted in a CO-saturated electrolyte to probe the surface species during CORR (0.01 M KOH and 0.045 M K_2_SO_4_, pH 11.7), **e** SEM image of Cu foil with Au@SiO_2_ (inset image: TEM image of Au@SiO_2_) reproduced with permission from [15]. Copyright (2020) American Chemical Society. In situ Raman spectra measured at various potentials on **f** Cu microparticle in CO-saturated 0.3 M NaH_2_PO_4_/0.35 M Na_2_HPO_4_ (pH 7.2). **g** Cu microparticle in CO-saturated 0.1 M NaOH/0.9 M NaClO_4_ (pH 12.9), reproduced with permission from [47]. Copyright (2021) Springer Nature

As shown in Fig. 7, Zhao et al. used SHINERS to unravel the surface species on Cu foils with and without Au@SiO_2_ during the reaction [15]. The in situ Raman spectra recorded without Au@SiO_2_ (Fig. 7a and c) showed no Raman signals from the Cu foil in the examined potential range of open circuit potential (OCP) to –0.8 V in the CO saturated electrolyte, except for a sulfate ion peak at 980 cm^−1^ originating from the electrolyte. However, the in situ Raman spectra recorded in the presence of Au@SiO_2_ (Figs. 7b and d) revealed multiple surface species related to Cu. At the OCP, two broad bands occurred at 490 and 600 cm^−1^ and were assigned to the partially reduced Cu_2_O phase (Cu_2_O_1−x_). At a more negative potential, a peak appeared at 612–622 cm^−1^. They confirmed that the peak corresponded to Cu–O_ad_ because there was no peak shifting from the isotopic substitution of H_2_O with D_2_O [15]. Additionally, as shown in Fig. 7d, there were additional peaks from the adsorbed CO on the Cu surface. These results demonstrate that SHINERS not only provide information on surface intermediates but also aid in the monitoring of phase evolution of catalyst surfaces during electrochemical reactions. The SEM image of Cu foil decorated with Au@SiO_2_ is shown in Fig. 7e. The inset picture is a TEM image of Au@SiO_2_ with a shell thickness of ~ 2 nm.

Meanwhile, Li et al. used SHINERS to investigate the effect of electrolytes on surface species and active sites on dendritic Cu microparticles [47]. Figure 7f–g show in situ Raman spectra of CO-saturated 0.3 M NaH_2_PO_4_/0.35 M Na_2_HPO_4_ and CO-saturated 0.1 M NaOH/0.9 M NaClO_4_. The author kept the sodium cation concentration (1 M) constant in all electrolyte compositions to avoid the cation effect in CORR activity comparison. Broad Raman bands at 528 and 618 cm^−1^ assigned to the Cu_2_O surface appeared at the OCP and in the phosphate-containing electrolyte and gradually decreased at increasingly negative potentials. At the potential region between – 0.2 and – 0.7 V vs. SHE, a weak band corresponding to Cu–O_ad_ was observed at 595 cm^−1^. At potentials below – 1.1 V vs. SHE, a Raman band assigned to a CuO_x_/(OH)_y_ mixed-phase appeared at ~ 530 cm^−1^. Additionally, two different bands (931 and 1152 cm^−1^) assigned to phosphates were identified at potentials below – 0.2 V vs. SHE. Interestingly, the band at 1152 cm^−1^ shifted toward a relatively low wavenumber as the potential became increasingly negative. This behavior, known as the Stark tuning effect, demonstrated that the phosphate was adsorbed on the catalyst surface [47]. Contrarily, there were no phosphate bands in the 0.1 M NaOH/0.9 M NaClO_4_ electrolyte. The strongest Raman signals were produced by CuO_x_/(OH)_y_ at ~ 530 cm^−1^ and ClO_4_^−^ in the bulk electrolyte at 935 cm^−1^. Based on these results, they proposed that the competitive adsorption of CO and phosphate affects the coverage and heterogeneity of the adsorbed CO, resulting in different CORR activities [47]. This study shows that the adsorption behavior of electrolyte species and their interactions with reaction intermediates can be observed by SHINERS.

## Carbon monoxide oxidation reaction

**Fig. 8:**
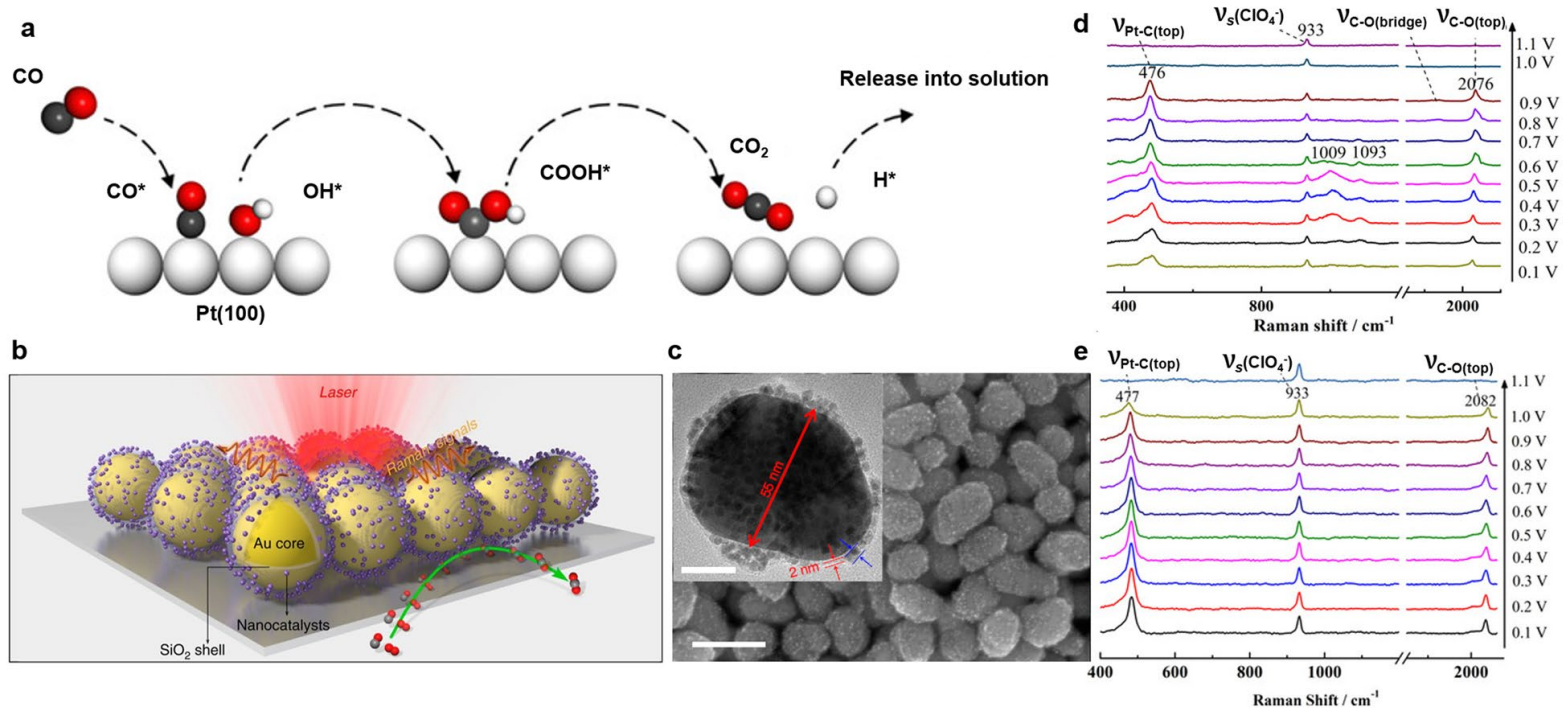
**a** The proposed mechanism of CO electrooxidation on the Pt(100) surface, reproduced with permission from [65]. Copyright (2020) Wiley-VCG GmbH. **b** The satellite structure composed of Au core/silica shells and nanocatalysts. High density of interfaces between the SHINs and nanocatalysts allow for high intensity of SERS signal. **c** The SEM images of the satellite structure (inset picture: TEM image of the satellite structure), reproduced with permission from [71]. Copyright (2017) Springer Nature Limited. SHINERS spectra of CO electrooxidation on the **c** Pt(100) and **d** Pt(110) surfaces in 0.1 M HClO_4_ solution saturated with CO, reproduced with permission from [65]. Copyright (2020) Wiley-VCG GmbH

CO has been reported to poison Pt catalyst surfaces during oxidation reactions in the anode of a fuel cell because it possesses a strong binding affinity with Pt surfaces, which can decrease the catalyst durability and performance [70]. Understanding the adsorption and oxidation of CO molecules is an important step in designing enhanced catalysts. According to previous studies [65], the Co electrooxidation reaction occurs through a surface bimolecular reaction in which the CO* and OH* intermediates adsorbed on the catalyst surface participate in the formation of COOH* intermediates, as shown in Fig. 8a. Thereafter, the COOH* intermediate is further oxidized into carbon dioxide and released into the solution.

The Tian group used SHINERS to understand the electrooxidation mechanism of CO on Pt(hkl) surfaces in acidic conditions [64, 71]. As shown in Fig. 8b and c, they developed a satellite structure using nanocatalysts deposited on Au@SiO_2_ nanoparticles. Probing nanocatalysts closely attached to the SiO_2_ shell can amplify the Raman signals from chemical species adsorbed on the catalyst surface. In situ Raman spectra were recorded in a 0.1 M HClO4 aqueous electrolyte over a potential range of 0.1–1.1 V [64, 71].

Figure 8d–e shows the peaks recorded at the initial potential (0.1 V), including the Pt–C stretching mode of CO absorbed on the bridge site (410 cm^−1^) and top site (480 cm^−1^), the symmetric stretching mode of the ClO_4_^−^ ion (933 cm^−1^), and the C–O stretching mode of CO absorbed on the bridge site (1780 cm^−1^) and top site (2050 cm^−1^). However, on Pt(100) surface at 0.2 and 0.3 V, two other Raman peaks appeared at 1090 cm^−1^ and 1005 cm^−1^, respectively. To confirm the assignment of the peaks, isotopic substitution with deuterium was conducted, and it was discovered that both peaks shifted to lower wavenumber. These results indicated the intermediate species responsible for the vibration contains a hydrogen atom. Furthermore, the DFT calculations revealed that the peak at 1005 cm^−1^ is assigned to the C–OH stretching vibration of the COOH* intermediate, while the peak at 1090 cm^−1^ is attributed to the bending mode of OH* absorbed on the Pt surface [65]. These SHINERS results provided improved insight into the CO adsorption and oxidation processes that occur during the anode reaction.

## Water oxidation reaction

The water-splitting reaction has been investigated as a promising solution for the generation of hydrogen energy from renewable sources. IrO_x_ is known as a water oxidation catalyst that has good stability under acidic conditions and the lowest overpotentials for OERs [46]. Although IrO_x_ has been studied for a long time, the OER mechanism for IrO_x_ remains still unknown.

**Fig. 9:**
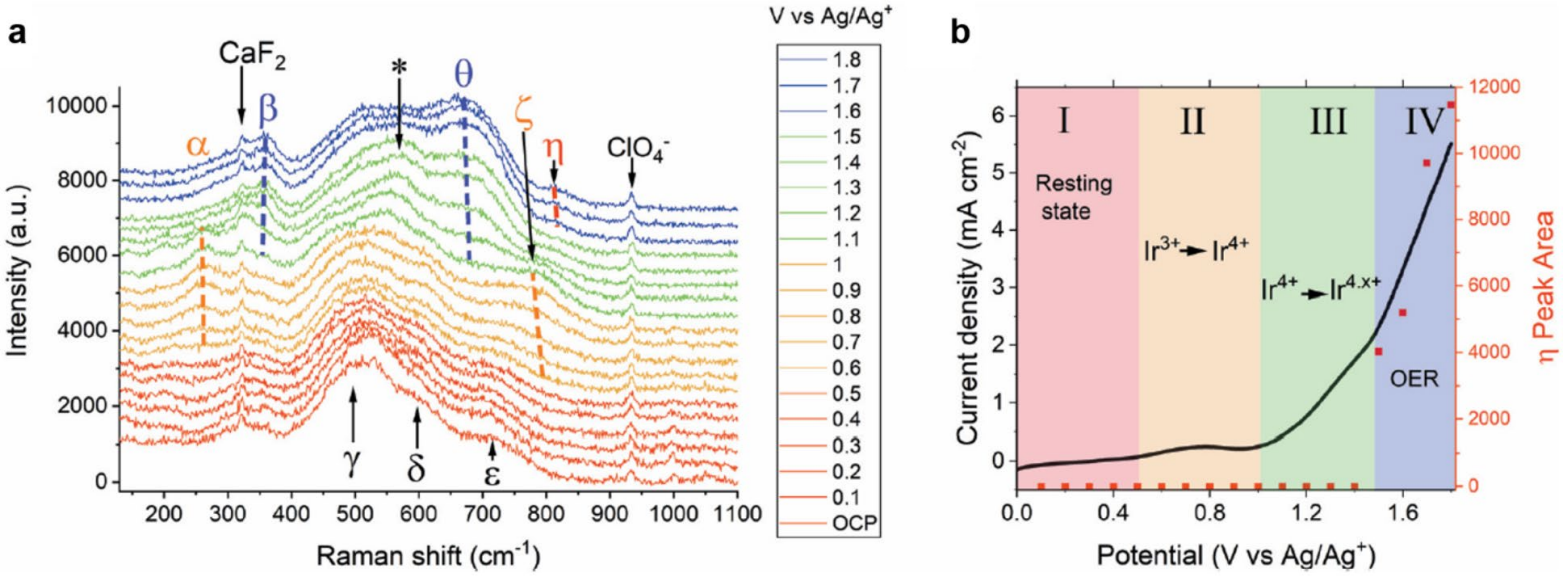
**a** In situ Raman spectra of an Au@SiO_2_ particle-coated IrO_x_ film measured in a 1 M NaClO_4_ (pH 10) electrolyte at various potentials. **b** OER current density and η peak area as a function of the potential. The voltammogram was measured at a scan rate of 5 mV s^−1^ in 1 M NaClO_4_ (pH 10) adjusted with NaOH, reproduced with permission from [46]. Copyright (2020) The Royal Society of Chemistry

Cowen’s group used SHINERS to investigate the water oxidation intermediates on IrOx electrodes in a 1 M NaClO_4_ electrolyte at pH 10 [46]. The potential was scanned from the OCP to 1.8 V vs. Ag/Ag^+^ potential sweep while recording the in situ Raman spectra (Fig. 9a). The result revealed that an iridium oxo intermediate (Ir = O) indicated by η was formed above 1.6 V. Furthermore, the Raman peak of the Ir = O species was discovered to have a relationship with the OER current density, as shown in Fig. 9b. The SHINERS result helped to experimentally support the OER mechanism of the proposed IrOx catalysts (see Table 2).

**Table 2:** Raman peak assignment of IrO_x_ films captured using the SHINERS technique (Fig. 9a) [46]

Label	Peak position [cm^−1^]	Assignment		Species
α	262	Ir–O–Ir twist		Ir^4+^
β	357	Ir–O–Ir twist		Ir^4.x+^
γ	504	Ir–O–Ir stretch		Ir^4+^
δ	608	Ir–O–Ir stretch		Ir^3+^
ε	719	Ir–O–Ir stretch		Ir^4+^
ζ	773	Ir–O–Ir stretch		Ir^4+^
θ	672	Ir–O–Ir stretch		Ir^4.x+^
η	813 in H2O	Ir=O stretch		Ir^4.x+^
	817 in D2O			

## Nitrate reduction reaction

Ammonia (NH_3_) is an essential chemical feedstock because of its wide applications, such as pharmaceuticals and fertilizers. It is also considered a hydrogen energy carrier for a carbon–neutral future. The Haber–Bosch process, which requires high temperatures and pressures, is mainly used to manufacture NH_3_ industrially. Industrial NH_3_ production causes a 1.44% increase in global CO_2_ emissions because of its harsh synthesis conditions [72]. Currently, the removal of nitrate from industrial wastewater and its electrochemical conversion into NH_3_ has been an important topic in the environmental research field. The Cu metal is one of the most active nitrate electrocatalysts; however, there is still a lack of understanding regarding the surface chemical species and their influence on the reduction activity on Cu surfaces [50].

**Fig. 10:**
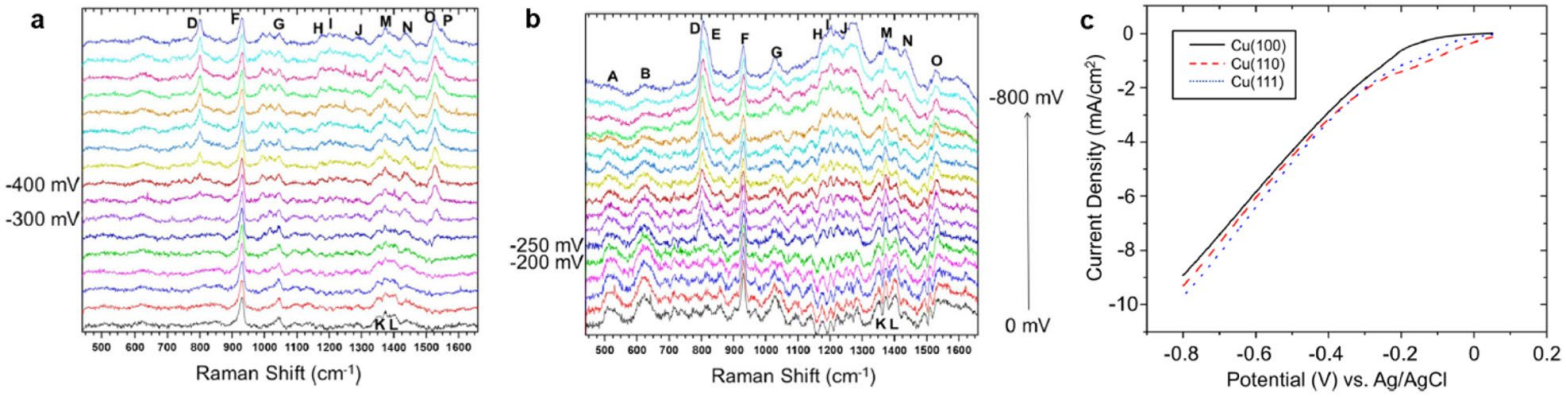
In situ Raman spectra of **a** Cu(100) and **b** Cu(110) electrodes measure in a 0.1 M HClO_4_/0.05 M HNO_3_ solution during cathodic polarization from 0 to 800 mV vs. Ag/AgCl. **c** Linear sweep voltammetry of Cu(100), Cu(110), and Cu(111) electrodes in a 0.1 M HClO_4_/0.05 M HNO_3_ solution during cathodic polarization from 0 to 800 mV vs. Ag/AgCl, reproduced with permission from [50]. Copyright (2016) Elsevier

The Gewirth group used SHINERS to investigate the active sites and reaction intermediates on nitrate reduction into NH_3_ for all Cu(hkl) surface with and without HCl addition (Fig. 10) [50]. They mentioned that the RDS is the initial two-electron reduction of nitrate into nitrite. The nitrous oxide species (mentioned with D, E, and G to O peaks in Fig. 10) have similar spectra on the Cu(100) (Fig. 10a) and Cu(11) surfaces (Fig. 10b), whereas Cu_2_O species (described with A and B letters) only occurred on the Cu(110) surface. Such a different active site was proposed to affect the rate-determining step for NH_3_ production, which is the two-electron reduction of NO_3_^−^ to NO_2_^−^. As shown in the linear sweep voltammetry (LSV) curves, the Cu(110) surface showed a more positive onset potential for nitrate reduction than the Cu(100) surface (Fig. 10c). Based on these results, SHINERS was able to confirm the role of the oxidized Cu surface in nitrate reduction (see Table 3).

**Table 3:** Raman peak assignment for the NO_3_RR on the Cu(100) and Cu(110) surfaces observed using the SHINERS technique [50]

Peak	Raman shift [cm^−1^]		Assignment
	**Cu(100)**	**Cu(110)**	
A		509	Cu_2_O
B		619	Cu_2_O
D	801	801	NO_2_¯ bending
E		817	NO_2_¯ bending
F	931	931	v(Cl-O)
G	1044	1027	V_s_ NO stretching
H	1180	1183	v_s_ NO_2_¯, chelating nitrito
I	1200	1208	v(N–O) nitrito orientation
J	1281	1272	v_a_ NO_2_¯, chelating nitrito
K	1350	1349	v_s_ NO_2_¯ of NO_3_¯
L	1372	1373	v_a_ NO_2_¯ of NO_3_¯
M	1372	1374	v(N = O), nitrito orientation
N	1434	1431	v(N = O), bridging nitro
O	1528	1531	v(N = O), nitroxyl
P			HNO bending and stretching, nitroxyl

## Conclusion

In summary, we review the fundamental principles and historical development of SHINERS, as well as discuss new insights into investigating reaction intermediates, catalytic active sites, and related reaction mechanisms using the SHINERS method. SHINERS is a critical strategy for capturing intermediate species of various electrochemical reactions even on smooth electrodes and metal oxide films despite low SERS activity, as demonstrated in the mechanistic studies for ORRs and COORs on different Pt single crystal surfaces. Simultaneously, SHINs can significantly amplify SERS signals from a thin layer of surface reactive sites. As shown in the case studies of water oxidation reactions, CORRs, and NO_3_RRs, SHINERS allows for monitoring of the potential-dependent behaviors of electrode surfaces and for determining the catalytically active sites by comparing the SHINERS results with chronoamperometric and linear sweep voltammetric measurements. Therefore, SHINERS can be used to investigate a reaction mechanism regardless of the type of electrode, and it has a wide potential in a variety of electrocatalysis and electrochemical systems. We believe that this review not only provides a comprehensive understanding of the SHINERS technique in unraveling reaction mechanisms for electrocatalysis but also provides new insights into observing catalyst–electrolyte interfaces that have not been probed.

## Data Availability

All data presented in this review article are included in the published article which can be found in the references list.

## References

[1] Burnham A, Han J, Clark CE, Wang M, Dunn JB, Palou-Rivera I (2012). Environ. Sci. Technol..

[2] Kennedy C, Steinberger J, Gasson B, Hansen Y, Hillman T, Havránek M, Pataki D, Phdungsilp A, Ramaswami A, Mendez GV (2009). Environ. Sci. Technol..

[3] Anderson TR, Hawkins E, Jones PD (2016). Endeavour.

[4] bp Statistical Review of World Energy (2020). https://www.bp.com/content/dam/bp/business-sites/en/global/corporate/pdfs/energy-economics/statistical-review/bp-stats-review-2020-full-report.pdf

[5] Sa YJ, Lee CW, Lee SY, Na J, Lee U, Hwang YJ (2020). Chem. Soc. Rev..

[6] Abbasi TASA (2011). Renew. Sustain. Energy Rev..

[7] She ZW, Kibsgaard J, Dickens CF, Chorkendorff I, Nørskov JK, Jaramillo TF (2017). Science.

[8] Askins EJ, Zoric MR, Li M, Luo Z, Amine K, Glusac KD (2021). Nat. Commun..

[9] Lee CW, Cho NH, Im SW, Jee MS, Hwang YJ, Min BK, Nam KT (2018). J. Mater. Chem. A.

[10] Lee CW, Kim C, Min BK (2019). Nano Converg.

[11] Chen J, Liu G, Zhu YZ, Su M, Yin P, Wu XJ, Lu Q, Tan C, Zhao M, Liu Z, Yang W, Li H, Nam GH, Zhang L, Chen Z, Huang X, Radjenovic PM, Huang W, Tian ZQ, Li JF, Zhang H (2020). J. Am. Chem. Soc..

[12] Zhu K, Zhu X, Yang W (2019). Angew. Chem. Int. Ed..

[13] Jiao W, Chen C, You W, Chen G, Xue S, Zhang J, Liu J, Feng Y, Wang P, Wang Y, Wen H, Che R (2020). Appl. Catal. B Environ..

[14] Deng Y, Yeo BS (2017). ACS Catal..

[15] Zhao Y, Chang X, Malkani AS, Yang X, Thompson L, Jiao F, Xu B (2020). J. Am. Chem. Soc..

[16] Moradzaman M, Mul G (2021). ChemElectroChem.

[17] Long C, Han J, Guo J, Yang C, Liu S, Tang Z (2021). Chem Catal..

[18] Chernyshova IV, Somasundaran P, Ponnurangam S (2018). Proc. Natl. Acad. Sci. USA.

[19] Li JF, Zhang YJ, Ding SY, Panneerselvam R, Tian ZQ (2017). Chem. Rev..

[20] Dong JC, Zhang XG, Briega-Martos V, Jin X, Yang J, Chen S, Yang ZL, Wu DY, Feliu JM, Williams CT, Tian ZQ, Li JF (2019). Nat. Energy.

[21] Li JF, Huang YF, Ding Y, Yang ZL, Li SB, Zhou XS, Fan FR, Zhang W, Zhou ZY, Wu DY, Ren B, Wang ZL, Tian ZQ (2010). Nature.

[22] Ding SY, Yi J, Li JF, Ren B, Wu DY, Panneerselvam R, Tian ZQ (2016). Nat. Rev. Mater..

[23] Lal S, Link S, Halas NJ (2007). Nat. Photon..

[24] S. Cong, X. Liu, Y. Jiang, W. Zhang, and Z. Zhao, The Innovation **1**, 3, 100051 (2020). 10.1016/j.xinn.2020.100051 PMC8454671 34557716

[25] Zuloaga J, Prodan E, Nordlander P (2009). Nano Lett..

[26] Tittl A, Yin X, Giessen H, Tian XD, Tian ZQ, Kremers C, Chigrin DN, Liu N (2013). Nano Lett..

[27] Zhang H, Duan S, Radjenovic PM, Tian ZQ, Li JF (2020). Acc. Chem. Res..

[28] Fleischmann M, Hendra PJ, McQuillan AJ (1974). Chem. Phys. Lett..

[29] Jeanmaire DL, Van Duyne RP (1977). J. Electroanal Chem..

[30] Moskovits M (1978). J. Chem. Phys..

[31] Kuruvinashetti K, Zhang Y, Li J, Kornienko N (2020). New J. Chem..

[32] Shafiqa AR, Abdul Aziz A, Mehrdel B (2020). J. Phys. Conf. Ser..

[33] Fang P-P, Li J, Yang Z-L, Li L-M, Ren B, Tian Z (2008). J. Raman Spectrosc..

[34] Cortijo-Campos S, Ramírez-Jiménez R, Climent-Pascual E, Aguilar-Pujol M, Jiménez-Villacorta F, Martínez L, Jiménez-Riobóo R, Prieto C, de Andrés A (2020). Mater. Des..

[35] Hlaing M, Gebear-Eigzabher B, Roa A, Marcano A, Radu D, Lai CY (2016). Opt. Mater. (Amst)..

[36] Benz F, Chikkaraddy R, Salmon A, Ohadi H, De Nijs B, Mertens J, Carnegie C, Bowman RW, Baumberg JJ (2016). J. Phys. Chem. Lett..

[37] Li SB, Li LM, Anema JR, Li JF, Yang ZL, Ren B, Sun JJ, Tian ZQ (2011). Zeitschrift. Fur. Phys. Chem..

[38] Kołątaj K, Krajczewski J, Kudelski A (2018). Appl. Surf. Sci..

[39] Krajczewski J, Michałowska A, Kudelski A (2020). Spectrochim. Acta Part A.

[40] Turkevich J, Stevenson PC, Hillier J (1951). Discuss. Faraday Soc..

[41] Frens G (1973). Nat. Phys. Sci..

[42] Sau TK, Murphy CJ (2004). J. Am. Chem. Soc..

[43] Quyen TTB, Chang CC, Su WN, Uen YH, Pan CJ, Liu JY, Rick J, Lin KY, Hwang BJ (2014). J. Mater. Chem. B.

[44] Li JF, Anema JR, Yu YC, Yang ZL, Huang YF, Zhou XS, Ren B, Tian ZQ (2011). Chem. Commun..

[45] Li JF, Li SB, Anema JR, Yang ZL, Huang YF, Ding Y, Wu YF, Zhou XS, Wu DY, Ren B, Wang ZL, Tian ZQ (2011). Appl. Spectrosc..

[46] Saeed KH, Forster M, Li JF, Hardwick LJ, Cowan AJ (2020). Chem. Commun..

[47] Li J, Chang X, Zhang H, Malkani AS, Jeng Cheng M, Xu B, Lu Q (2021). Nat. Commun..

[48] Galloway TA, Hardwick LJ (2016). J. Phys. Chem. Lett..

[49] Huang YF, Kooyman PJ, Koper MTM (2016). Nat Commun.

[50] Butcher DP, Gewirth AA (2016). Nano Energy.

[51] Sanchez-Gaytan BL, Swanglap P, Lamkin TJ, Hickey RJ, Fakhraai Z, Link S, Park SJ (2012). J. Phys. Chem. C.

[52] Kelly KL, Coronado E, Zhao LL, Schatz GC (2003). J. Phys. Chem. B.

[53] Rycenga M, Langille MR, Personick ML, Ozel T, Mirkin CA (2012). Nano Lett..

[54] Zhang Q, Large N, Wang H, Appl ACS (2014). Mater. Interfaces.

[55] Lee JH, Gibson KJ, Chen G, Weizmann Y (2015). Nat Commun.

[56] Keunen R, MacOretta D, Cathcart N, Kitaev V (2016). Nanoscale.

[57] Uzayisenga V, Lin XD, Li LM, Anema JR, Yang ZL, Huang YF, Lin HX, Li SB, Li JF, Tian ZQ (2012). Langmuir.

[58] Wiley B, Sun Y, Xia Y (2007). Acc. Chem. Res..

[59] Botella P, Ortega Í, Quesada M, Madrigal RF, Muniesa C, Fimia A, Fernández E, Corma A (2012). Dalt. Trans..

[60] Liz-marza LM, Giersig M, Mulvaney P (1996). Langmuir.

[61] Li JF, Tian XD, Li SB, Anema JR, Yang ZL, Ding Y, Wu YF, Zeng YM, Chen QZ, Ren B, Wang ZL, Tian ZQ (2013). Nat. Protoc..

[62] Zhang W, Dong JC, Li CY, Chen S, Zhan C, Panneerselvam R, Yang ZL, Li JF, Zhou YL (2015). J. Raman Spectrosc..

[63] Acharya A, Dubbu S, Kumar S, Kumari N, Kim Y, So S, Kwon T, Wang Z, Park J, Cho YK, Rho J, Oh SH, Kumar A, Lee IS (2021). J. Am. Chem. Soc..

[64] Liang MM, Wang YH, Shao R, Yang WM, Zhang H, Zhang H, Yang ZL, Li JF, Tian ZQ (2017). Electrochem. Commun..

[65] Su M, Dong JC, Le JB, Zhao Y, Yang WM, Yang ZL, Attard G, Liu GK, Cheng J, Wei YM, Tian ZQ, Li JF (2020). Angew. Chem. Int. Ed..

[66] Lee CW, Yang KD, Nam DH, Jang JH, Cho NH, Im SW, Nam KT (2018). Adv. Mater..

[67] Greeley J, Stephens IEL, Bondarenko AS, Johansson TP, Hansen HA, Jaramillo TF, Rossmeisl J, Chorkendorff I, Nørskov JK (2009). Nat. Chem..

[68] Wang Y, Le J, Li W, Wei J, Radjenovic PM, Zhang H, Zhou X, Cheng J, Tian Z, Li J (2019). Angew. Chem..

[69] Dong JC, Su M, Briega-Martos V, Li L, Le JB, Radjenovic P, Zhou XS, Feliu JM, Tian ZQ, Li JF (2020). J. Am. Chem. Soc..

[70] Lee CW, Shin SJ, Jung H, Nguyen DLT, Lee SY, Lee WH, Won DH, Kim MG, Oh HS, Jang T, Kim H, Min BK, Hwang YJ (2019). ACS Energy Lett..

[71] Zhang H, Wang C, Sun HL, Fu G, Chen S, Zhang YJ, Chen BH, Anema JR, Yang ZL, Li JF, Tian ZQ (2017). Nat. Commun..

[72] Wu ZY, Karamad M, Yong X, Huang Q, Cullen DA, Zhu P, Xia C, Xiao Q, Shakouri M, Chen FY, Timothy Kim JY, Xia Y, Heck K, Hu Y, Wong MS, Li Q, Gates I, Siahrostami S, Wang H (2021). Nat. Commun..

